# Resolving the discrepancies of suicide risk in obsessive-compulsive patients: a review of incidence rates and risk factors of suicide and suicide attempts in OCD

**DOI:** 10.1192/bjo.2021.787

**Published:** 2021-06-18

**Authors:** Himanshu Tyagi, Gabriel Bundies

**Affiliations:** 1 University College London Hospitals NHS Trust, National Hospital for Neurology and Neurosurgery, University College London Institute of Neursology; 2 University College London

## Abstract

**Aims:**

Obsessive-Compulsive Disorder (OCD) describes a mental health condition in which affected patients experience persistent obsessions, which may often, but not always result in compulsions, causing major distress and anxiety. Obsessions are defined as intrusive thoughts with a high emotional valence, whereas compulsion are repetitive actions, which demonstrate the attempt to eliminate obsessive thoughts.

When speaking of OCD, risk of suicide is rarely a topic of concern. There is still no consensus about whether OCD and suicide are associated. Early schools of clinical sciences propose a low risk of harm, which was taught to most mental health practitioners currently working in health services. Moreover, the World Health Organisation currently classifies OCD as the 11th leading cause of nonfatal burden, indicating that despite the potential for causing significant disabilities, OCD does not pose any serious health risks. Contemporary evidence, however, suggests that the risk for suicide may be underestimated.

This literature review aims to cumulate evidence for the risk of suicide in OCD and its associated underlying factors to clarify and resolve the discrepancies that currently exist regarding this topic.

**Method:**

To identify eligible studies, the databases MEDLINE(R), PubMed, and PsycINFO are used. Selected studies provide data on suicide rates, attempts, and risk factors. Grey literature is included in the review to consider results from studies which may not have qualified for publishing. This literature review is conducted according to the PRISMA guidelines.

**Result:**

After deduplication, 653 studies could be found out of which 15 studies meet the inclusion criteria. Rates of suicide attempts appear to lie between 12% and 27%. Death-to-suicide rates in OCD are shown to range from 0.7% to 1.4%. Associated risk factors for suicide in OCD include, mistrust and unacceptable thoughts, depression, and comorbid substance use disorders. The strongest predictor for death caused by suicide is having a history of previous suicide attempts. Higher education and comorbid anxiety disorders act as protective factors. Lastly, gender differences remain unclear since some studies classify female sex as a protective, and some as a risk factor.

**Conclusion:**

This review provides a good overview of the actual risk for suicide in OCD. Current evidence suggests high suicidality in patients with OCD, leading to suicide attempts in affected patients, but not necessarily resulting in death, as the death-to-suicide rates are low. Genetic heritage and comorbidities of further mental health disorders may increase the risk for suicide in OCD.

